# Phenotypical Analysis of Atypical PKCs *In Vivo* Function Display a Compensatory System at Mouse Embryonic Day 7.5

**DOI:** 10.1371/journal.pone.0062756

**Published:** 2013-05-14

**Authors:** Sebastian Seidl, Ursula Braun, Norbert Roos, Shaohua Li, Timo H.-W. Lüdtke, Andreas Kispert, Michael Leitges

**Affiliations:** 1 The Biotechnology Centre of Oslo, University of Oslo, Oslo, Norway; 2 Department of Molecular Biosciences, University of Oslo, Oslo, Norway; 3 Department of Surgery, University of Medicine and Dentistry of New Jersey–Robert Wood Johnson Medical School, New Brunswick, New Jersey, United States of America; 4 Institute for Molecular Biology, Medizinische Hochschule Hannover, Hannover, Germany; Konkuk University, Republic of Korea

## Abstract

**Background:**

The atypical protein kinases C (PKC) isoforms ι/λ and ζ play crucial roles in many cellular processes including development, cell proliferation, differentiation and cell survival. Possible redundancy between the two isoforms has always been an issue since most biochemical tools do not differentiate between the two proteins. Thus, much effort has been made during the last decades to characterize the functions of aPKCs using gene targeting approaches and depletion studies. However, little is known about the specific roles of each isoform in mouse development.

**Methodology/Principal Findings:**

To evaluate the importance of PKCι in mouse development we designed PKCι deletion mutants using the gene targeting approach. We show that the deletion of PKCι, results in a reduced size of the amniotic cavity at E7.5 and impaired growth of the embryo at E8.5 with subsequent absorption of the embryo. Our data also indicate an impaired localization of ZO-1 and disorganized structure of the epithelial tissue in the embryo. Importantly, using electron microscopy, embryoid body formation and immunofluorescence analysis, we found, that in the absence of PKCι, tight junctions and apico-basal polarity were still established. Finally, our study points to a non-redundant PKCι function at E9.5, since expression of PKCζ is able to rescue the E7.5 phenotype, but could not prevent embryonic lethality at a later time-point (E9.5).

**Conclusion:**

Our data show that PKCι is crucial for mouse embryogenesis but is dispensable for the establishment of polarity and tight junction formation. We present a compensatory function of PKCζ at E7.5, rescuing the phenotype. Furthermore, this study indicates at least one specific, yet unknown, PKCι function that cannot be compensated by the overexpression of PKCζ at E9.5.

## Introduction

The protein kinase C (PKC) family of serine-threonine kinases consists of 9 different genes giving rise to at least 12 isoforms, subdivided into 3 subfamilies. The subdivision is based on sequence homology as well as dependency on cofactors during their activation process. Thus the classical PKCs (cPKC: α, β and γ) are dependent on Ca^2+^ ions and diacylglycerol for their activation whereas the novel PKCs (nPKC: δ, ε, η and θ) based on the lack of a binding domain are Ca^2+^ ion independent but still require diacylglycerol for their activation. In sharp contrast, the subfamily of atypical PKCs (aPKC: ζ and ι/λ) are independent of Ca^2+^ ions and diacylglycerol for their activation, thereby, representing quite a distinguishable subgroup among the PKC family. This becomes even more pronounced by the fact that aPKCs are unresponsive to Tumor Promoting Agent 12-O-Tetradecanoylphorbol-13-Acetate (TPA) treatment, an early described feature of PKC family members (see review [1]). Nevertheless, all PKC family members (including aPKCs) have been described to participate in a multitude of signaling pathways involved in cell growth, differentiation and apoptosis.

In 1989 Nishizuka and coworkers isolated the PKCζ isoform from a rat cDNA library [2]. Four years later a second aPKC isoform (PKCι) was isolated and characterized [3] followed by the identification of the mouse ortholog PKC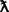 [4]. As true for all other PKCs, the aPKC proteins can be divided into a regulatory N-terminal region and a catalytic C-terminal region, separated by a hinge region. The exception is PKMζ which represents only the C-terminal kinase domain generated by an internal promotor whose activity has been assigned to neurons [5]. In contrast to other PKCs the N-terminal domain contains a Phox/Bem1 (PB1) motif mediating interaction with p62 [6], [7]and other signaling molecules like Mek5 [8] and Par6 [9] all of which are supposed to mediate aPKC signaling. In addition both aPKC also contain a cystein-rich zinc-finger like domain within the regulatory N-terminal domain defined as C1. Whereas all other PKCs possess a tandem repeat, aPKCs possess only one C1 domain. Interestingly, this domain accounts for the binding of diacylglycerol and TPA in classical and novel PKCs. The aPKC C1 domain has been reported to bind directly to phosphatidylinositol(3, 4, 5)-trisphosphat thereby inducing conformational changes in the protein leading to activation similar to diacylglycerol binding to other PKCs [10]. But also other interacting partners, inhibitory as well as activating, have been described [11], [12]. The aPKC C-terminal part represent the catalytic kinase domain sharing 86% homology to each other but only 45–55% to other PKCs. Overall both aPKCs show a 72% homology on amino acid level [4]. Due to high degree of homology and the limited availability of isoform-specific tools the *in vivo* analysis of isoform-specific aPKC functions remained insufficient in mammals. Nonetheless, it has been shown that aPKCs are conserved in numbers of organisms, including *C. elegans* [13] and *D. melanogaster* [14] in which only one isoform was detected.

We have previously shown that both aPKCs are expressed during mouse embryonic development [15] as well as in distinct domains in the adult mouse brain [16]. As a conclusion of these studies PKCι was defined as being ubiquitously expressed whereas PKCζ expression was pronounced in lung, kidney and brain. The spectrum of physiological processes linked to aPKCs function is huge and covers cell proliferation [17], cell polarity [18], carcinogenesis, neurogenesis [19] and many more. Attempts to investigate individual *in vivo* functions also made use of the gene targeting approach. We and other have generated aPKC deficient mouse lines which were subjected to various phenotypical investigations. Interestingly, the phenotype of the conventional PKCζ knockout did not display the expected phenotype during mouse preimplantation development [20]. Early studies using the conventional PKCζ knockout revealed a functional link to NFkB signaling [21], [22]. Subsequent studies also identified PKCζ to act as tumor suppressor due to its regulatory function on the IL-6 promotor [23]. In sharp contrast a conventional aPKCι knockout displayed an embryonic lethal phenotype [24] (personal communication with Shigeo Ohno and own data presented within this publication), clearly distinguishing both aPKC isoforms for the first time. Later studies using the conditional gene targeting approach showed inter alia specific *in vivo* functions for PKCι but not for PKCζ in muscle and podocytes [25], [26].

Atypical PKCs have been described to form complexes with the partition defective proteins Par6 and Par-3 [27]. Par genes have been cloned and characterized in 1995 [28] and were shown to be crucial for asymmetric cell division in *C. elegans* and other organisms [29], [30]. The association of aPKCs to this ternary complex, also called polarity complex [31] integrates aPKC signaling into all aspects of polarity without any isoform specificity.

Here, we subjected several established mutant mouse lines of the PKCι gene to a thorough developmental investigation. We present a detailed description of the embryonic lethal phenotype caused by the PKCι deficiency and provide more insights into the redundancy within the aPKC subfamily.

## Results

### Generation of 2 Different Prkci Knock Out Alleles in Mice

To investigate PKCι *in vivo* function, we decided to generate various mutant alleles following standard gene targeting approaches in mouse embryonic stem cells (ESCs). First we generated a conventional Prkci knock-out allele by inserting a neo cassette into the second exon of the gene causing a disruption of gene transcription (PKCιNeo, details see Figure S1A and material & methods). Since homozygozity of this allele caused embryonic lethality (see later), we decided to generate a conditional allele for Prkci using the Cre/loxP system in addition. For this approach the Prkci gene was targeted by inserting a single loxP site 5′prime of the 2nd exon followed by a floxed neo cassette 3′prime of the exon. This so-called floxed allele (PKCιflox/flox) has been described earlier and its functionality has previously been shown [25], [26], [32], [33]. In this study we subsequently crossed PKCιflox/flox mice with a transgenic mouse line [34] expressing the Cre recombinase in the germ line, in a ubiquitous fashion, starting at embryonic day (E) 13.5 and obtained a second null allele for Prkci named PKCιΔ(details see Figure S1B).

**Figure 1:**
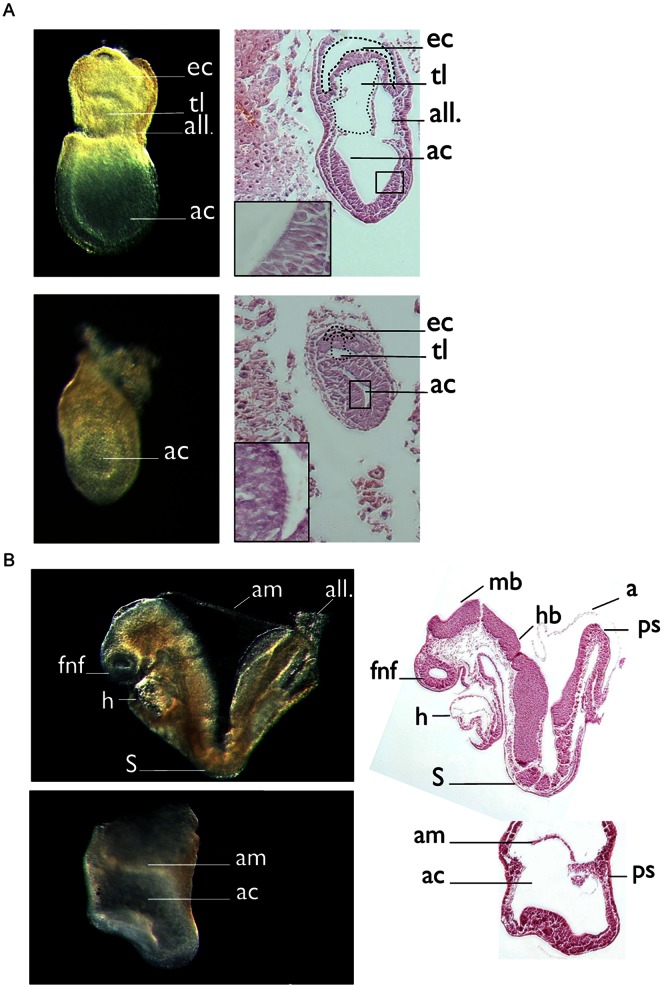
Histological overview of E7.5 and E8.5 embryos. Embryos were isolated at indicated time points and embedded in paraffin. (A) Sagittal sections of wt and PKCι^Δ/Δ^ embryos at E7.5 (right). Phase contrast image of the corresponding embryo (left) (B) Sagittal sections of wt and PKCι^Δ/Δ^ embryos at day E8.5 (right). Phase contrast image of the corresponding embryo (left). Abbreviations: ac, amniotic cavity; all, allantois; am, amnion; ec, ectoplacental cavity; fnf, forebrain neural-fold; hb, hindbrain; mb, midbrain; h, heart; ps, primitive streak; S = somites; tl; tiny lumina.

To investigate whether homozygozity of Prkci null alleles (PKCιNeo/Neo and PKCιΔ/Δ) are compatible with embryonic development, we analyzed more than 100 offspring from matings of heterozygous carries for each line at 3 weeks after birth. For both individual lines we did not observe any homozygous offspring whereas heterozygous (66%) and wt (33%) mice were detected (with an average of 8 pups per litter). These findings implied an embryonic lethal phenotype for both mutant alleles in agreement with earlier data published by Hedrick and coworkers [24]. In addition we also haveńt been able to observed any abnormalities in heterozygous animals within the first three month after birth. We next examined litters from heterozygous crosses at different developmental stages to identify the time point when first morphological abnormalities manifested in homozygous embryos. At embryonic day (E) 6.5, no morphological differences between litter-mates were apparent. At day E7.5, morphological inspection recognized a group of embryos which appeared “compressed” along the proximal-distal axis of the egg cylinder and harbored a small amniotic cavity (Figure 1A). Histological analysis showed that the epiblast was present but was thickened compared to non-affected litter-mates. Genotyping of individual embryos confirmed that this phenotype was restricted to homozygous embryos (25% of the investigated embryos of both transgenic lines showed this morphological abnormalities at this stage). At E8.5, mutant embryos were severely growth retarded. An anterior-posterior polarity of the body axis was not readily apparent since head folds and a linear heart tube distinguishing the anterior end of the embryo and the allantois representing the most posterior aspect were reduced or lacking (Figure 1B). In contrast to control embryos that harbored 4–6 somites at this stage, somites were not discernible by histological analysis in the mutants. By E10.5, mutant embryos were reabsorbed by the mother. Since phenotypic changes were indistinguishable between the two targeted alleles, we decided to exclusively use the PKCι△/△ line for further analysis.

### PKCι Embryos are Normally Patterned Along the Anterior-posterior Body Axis but Exhibit Defects in Mesodermal Differentiation

**Figure 2:**
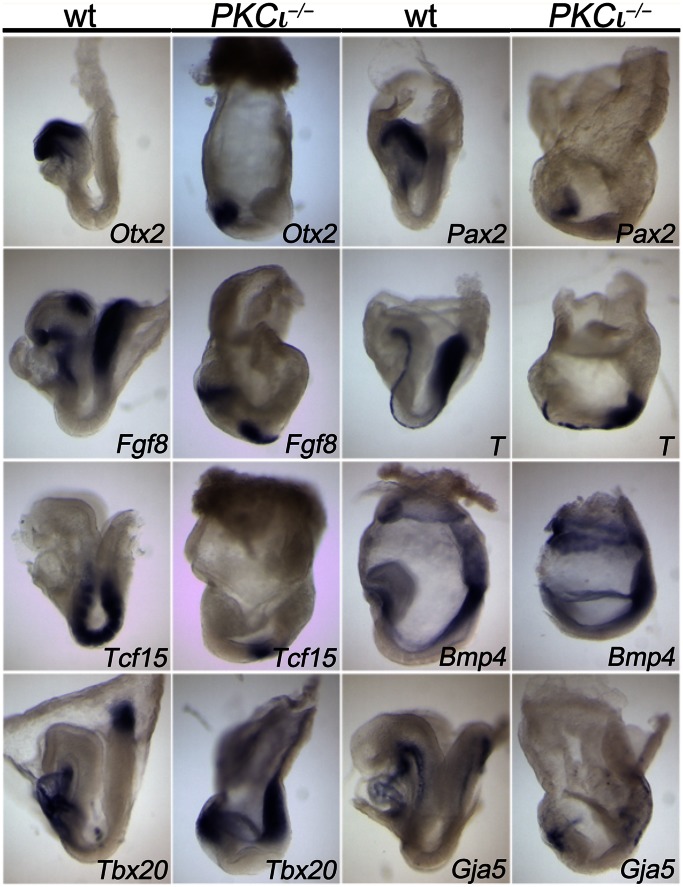
Whole-mount in situ hybridization analysis reveals an anterior-posterior body axis but a lack of a cardiovascular system in E8.5 embryos. Either wt (wt) or PKCι*-* deficient embryos were analyzed using a set of different markers: Otx2, orthodenticle homeobox 2; Fgf8, fibrobalst growth factor 8; Tcf15, transcription factor 15; Tbx20, T- box 20; Pax2, paired box gene 2; T, Brachyury; Bmp4, bone morphogenetic protein 4; Gja5, gap junction alpha-5 protein.

Since morphological and histological analysis indicated a deficit in axial elongation and germ layer formation, we used a panel of marker genes to test for formation and differentiation of the germ layers and establishment of an anterior-posterior body axis in E8.5 embryos (Figure 2).

In wt embryos, *Otx2* expression marks the forebrain [35], *Pax2* the midbrain [36] and *Fgf8* the midbrain/hindbrain boundary [37]. In PKCι-deficient embryos all three genes were expressed in the “anterior” embryonic region but the domains were compressed indicating that the (neuro-) ectoderm is patterned along the anterior-posterior axis but the expansion of the different primordial brain segments is compromised. *Fgf8* is additionally expressed in the primitive streak at the posterior end of the embryo overlapping with Brachyury (T) that additionally marks the notochord, a thin line of cells extending from the forebrain/midbrain border to the primitive streak underneath the neural tube [37], [38]. Expression of both genes was established in the mutant but the primitive streak appeared much reduced and the notochord was disrupted. This shows that PKCι mutant embryos are polarized along the anterior-posterior body axis but that differentiation of the axial mesoderm is impaired. *Tcf15* (also known as paraxis) marks the paraxial mesoderm [39] which was organized into 4–8 distinct somite pairs at this stage in the wt. In PKCι-deficient embryos a domain of *Tcf15* expression was found in the middle region of the embryos but individual somites were not formed. Expression of Bmp4 in the lateral plate mesoderm, the extraembryonic mesoderm and the extraembryonic ectoderm was found in mutant embryos as in the wt. *Tbx20* was expressed in the allantois, a derivative of the extraembryonic mesoderm, and in the linear heart tube, which derives from the cardiac subregion of the anterior lateral plate mesoderm in the wt [40]. Again, both expression domains were established at opposite poles of the mutant embryos but neither domain was appropriately developed into an allantois. Finally, *Gja*, that is expressed in the cardiac endoderm and dorsal aorta in wt embryos [41] was restricted to cell clusters at the anterior and posterior pole of the mutant embryos but clearly lacked an organization into vessel like structures. Together, this analysis suggests that PKCι-deficient embryos exhibit a normal anterior-posterior polarization of their main body axis. Mesoderm formation is initiated but comes to a premature halt, mesoderm differentiation into axial, paraxial, lateral and extraembryonic subtypes occurs but subsequent formation of tissues and organs completely fails. As a consequence PKCι-deficient embryos die due to lack of a cardiovascular system.

### Embryoid Body Formation is Impaired by PKCι Deficiency

ESCs, when cultured as aggregates, form spherical structures which are defined as embryoid bodies (EBs). These structures are thought to recapitulate early steps of the pre-implantation development including endoderm formation, basement membrane (BM) assembly, epiblast polarization and subsequent cavity formation [42]. When cultured in suspension for 5 days, EBs form epithelial cysts consisting of an outer endoderm and an inner columnar epiblast epithelium (CEE), separated by a BM. As PKCι mutants displayed an abnormal amniotic cavity at E7.5 we decided to use the embryoid body formation assay to analyze cavity formation in more detail.

**Figure 3:**
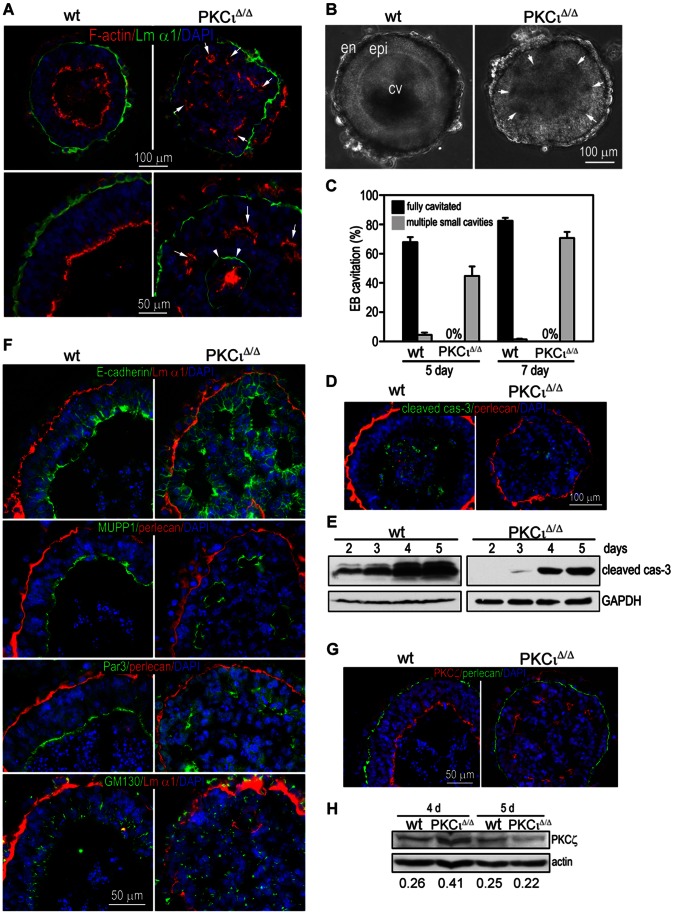
Immunofluorescence analysis of embryoid bodies (EBs) from either wt or PKCι^Δ/Δ^. (A) EBs from WT and PKCι^Δ/Δ^ ES cells were stained with Rhodamin-Phalloidin (red, F-actin) and an antibody against Laminin-α1 (green, Lm α1). (B) Phase-contrast picture of 5 days old WT and PKCι^Δ/Δ^ EBs. (C) Statistical analysis of lumen formation of 5 days and 7 days old EBs from WT and PKCι^Δ/Δ^ ES cells. (D) EBs from WT and PKCι^Δ/Δ^ ES cells were stained with antibodies against cleaved caspase-3 (green, cleaved cas-3) and perlecan (red). (E) WB analysis of activated caspase-3 at different time-points in WT and PKCι^Δ/Δ^ EBs. (F) Immunofluorescence analysis of EBs from either WT or PKCι^Δ/Δ^ ES cells including localization of E-cadherin, MUPP1, Par-3 and GM130 (green), perlecan and laminin α1 (red) (G) EBs from WT and PKCι^Δ/Δ^ ES cells were stained with antibodies against perlecan (green) and aPKCzeta (red, PKCζ). (H) WB analysis of PKCζ in 4 days and 5 days old WT and PKCι^Δ/Δ^ EBs. All membranes were scanned and the intensities of the single bands were calculated. Values represent rations of PKCζ to actin. Nucleus staining was performed with DAPI. Scale is as indicated.

When wt and *PKCι* deficient ESCs [43]were subjected to this assay, we detected obvious differences among the two genotypes. Wt EBs displayed the expected appearance in a phase contrast representation, PKCι^Δ/Δ^ EBs showed a disorganized structure (Figure 3 B). Most of the wt EBs formed a single large cavity enclosed by CEE after 5 days, whereas the mutant EBs did not form a single cavity at all or formed multiple small cavities adjacent to the polarized CEE, although the endoderm differentiated normally. When cultured for additional 2 days, most mutant EBs formed small cavities but failed to complete cavitation (Figure 3A, B and C). Since caspase-dependent apoptosis has been described as a key mechanism involved in the early steps of cavity formation [42] we immunostained 5-day EBs for cleaved (activated) caspase-3. Wt EBs displayed massive central apoptosis as evidenced by cleaved caspase-3 staining (Figure 3 D). By contrast, only scattered apoptotic cells were detected in PKCι deficient EBs. DAPI staining of condensed and fragmented nuclei, another hallmark of apoptosis, also showed reduced apoptosis at the center of mutant EBs (Figure 3D). To further analyze the apoptosis during EB cavitation, we cultured EBs of both genotypes for 2 to 5 days and performed immunoblotting for cleaved caspase-3. Indeed, PKCι deficient EBs expressed less apoptotic activity at all time points tested than the corresponding wt extracts (Figure 3E). These results demonstrate that PKCι deficient EBs fail to form a single cavity possibly due to reduced central apoptosis.

Gene targeting experiments have shown that laminin-mediated BM formation is essential for primitive ectoderm epithelialization and cavitation in EBs [44], [45]. The laminin α-1 chain is a key component of the embryonic BM expressed early on during development [46]. In the wt EB the laminin α-1 staining formed a continuous thin layer between endoderm and epiblast cells (Figure 3A). We did not observe obvious changes in BM assembly in mutant EBs. These findings were confirmed by using perlecan as an alternative marker for the BM (Figure 3F and G). However, ectopic BM formation was detected at the center of ∼20% mutant EBs (Figure 3F). This may be caused by failure in endodermal cell migration to the EB surface. Nonetheless, these results suggest that the cavitation defect in PKCι deficient EBs is unlikely caused to ectopic BM assembly.

Atypical PKCs have been functionally linked to the establishment and maintenance of epithelial polarity and tight junction formation due to their association with Par-3 and Par6 (forming the so-called polarity complex). To determine if epiblast polarity is disrupted in the absence of PKCι, we immunostained EBs for the apical marker MUPP1, Par-3, the adherens junction protein E-cadherin and the Golgi marker GM130. The apical actin belt was visualized using rhodamine-phalloidin. As shown in Figure 3A, F-actin staining was detected as a continuous layer at the most inner cell membrane surrounding the single cavity in wt EBs. In mutant EBs, no such staining was detectable. The apical markers MUPP1 and Par-3 were expressed at the same location (Figure 3F). The Golgi marker GM130 was positioned away from the BM and localized on the apical side. Similarly, E-cadherin was enriched at the apex of the epiblast where they formed AJs. These results provide evidence that polarity of the epiblast was intact.

Since the embryo and the EB express both PKCι and PKCζ, we were wondering if the remaining PKCζ could possibly compensate for some PKCι function. Therefore, we immunostained aPKC using a pan aPKC antibody in wt and mutant EBs. We were able to detect the protein at the apex of the epiblast of both genotypes (Figure 3G). Whether the signal in the wt reflects a combination of both aPKC isoforms is unclear but for sure the observed signal in the mutants represents PKCζ. In addition we also carried out a western blot analysis for aPKCs using protein extracts from EBs after 4 and 5 days of differentiation. The aPKC signal (ergo PKCζ in the PKCι deficient background) appeared slightly up-regulated at 4 days in PKCι deficient extracts whereas after 5 days it became down-regulated when compared with wt EBs (Figure 3H). Taken together these results suggest that PKCζ may compensate for the loss of PKCι to confer an apical-basal polarity to epithelial cells in the epiblast.

### Immunohistochemical and Electron Microscopical Investigation Revealed Subtle Changes in the Overall Organization of PKCι Deficient Embryos

Since PKCι deficient EBs lacked apoptosis, we wished to evaluate the contribution of this process in the mutant embryos as well. However, we failed to detect increased apoptosis by cleaved (activated) caspase-3 staining nor did we detect changes in cell proliferation using a phospho-specific histone 3 antibody between E7.5 embryos of either genotype (Figure S2).

**Figure 4:**
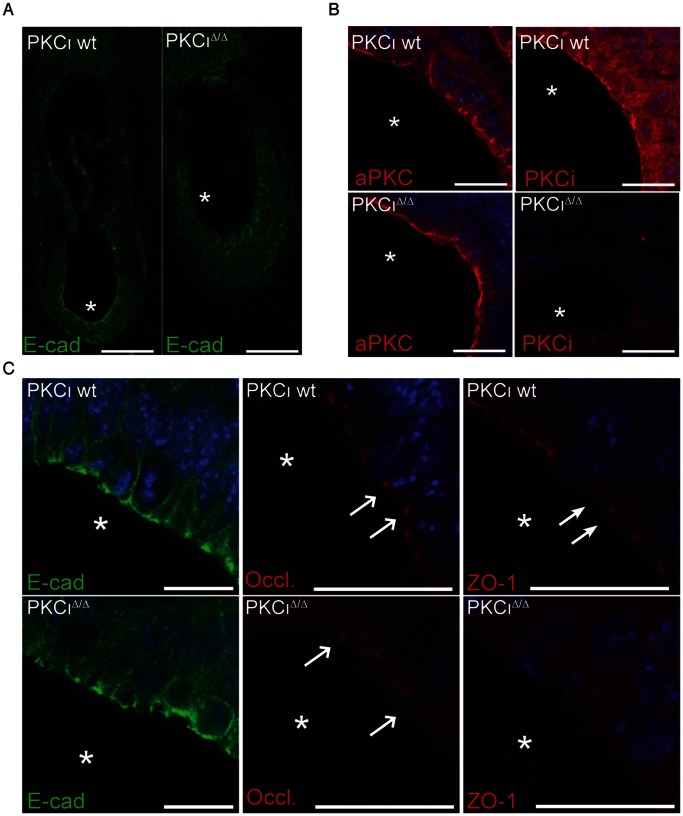
Immunofluorescence analysis of marker proteins in PKCι^Δ/Δ^ embryos. Analysis were performed using the indicated antibodies. (A) Paraffin sections showing the localization of E-cadherin (E-cad) in the wt and PKCι^Δ/Δ^ embryo. Scale bars: 100 µm (B) Immunofluorescence analysis of aPKC localization at the apical pole of wt and PKCι deficient embryos in high magnification. Scale bars: 20 µm (C) Immunofluorescence analysis of proteins involved in cellular polarity (E-cad, E-cadherin; Occl., occludin; ZO-1, Zonula Occludens-1). Localization of occludin is indicated by open arrows and the localization of ZO-1 by solid arrows. Scale bars: 20 µm.

We next analyzed whether the embryonic ectoderm of E7.5 mutant embryos harbored epithelial cells with normal apical-basal polarity and junctional complexes. Again we used an antibody against E-cadherin as a marker for adherens junctions (AJs). As shown in the overview and the higher magnification (Figure 4A and C) both genotypes displayed a clear signal at the apical side of the embryonic ectoderm. This indicated that polarity in the epithelial cell layer was established. However, in mutant embryos the signal did not form a sharp thin line but was interrupted at the apical side (Figure 4A and C). We then further applied occludin and zonula occludens-1 protein (ZO-1) specific antibodies as marker proteins for tight junctions (TJ). In both cases the wt showed a punctuated pattern at the apical pole as expected (Figure 4C, white arrows). In PKCι mutants we detected signals for occludin at the apical pole of the epithelial cell layer, but weaker (Figure 4C, open arrows). In contrast we were not able to detect any signal for ZO-1 at the apical side in mutants. Instead we observed a weak cytoplasmic background signal. A semi- quantitative RT-PCR analysis of E7.5 embryos did not amplify a significant ZO-1 specific transcript. Western blot analysis did not detect ZO-1 protein in at E8.5 PKCι mutants either (Figure S3). Thus we concluded that TJ exists in mutant embryos but that the composition of tight junction protein complexes is altered.

Based on our observation in EBs, we analyzed the aPKC content and localization in wt and mutant embryos. As shown in Figure 4B a commonly used antibody against aPKCs (Santa Cruz # sc121 C-20) stained primarily the apical pole of the embryonic ectoderm cell layer with a minor fraction also detected in the cytoplasm in the wt. In the mutant embryo the signal (representing PKCζ) is maintained at the apical side. When applying a PKCι specific antibody [47] the signal in the wt appeared broader, indicating that PKCι is more widely distributed within the cells than indicated by the pan aPKC antibody, but still showing an accumulation at the apical membrane. The PKCι mutant embryo did not show staining proving the specificity of the antibody. Hence, both aPKCs showed overlapping localization at the apical pole of embryonic ectodermal cells at E7.5 suggesting possible compensatory mechanisms.

**Figure 5:**
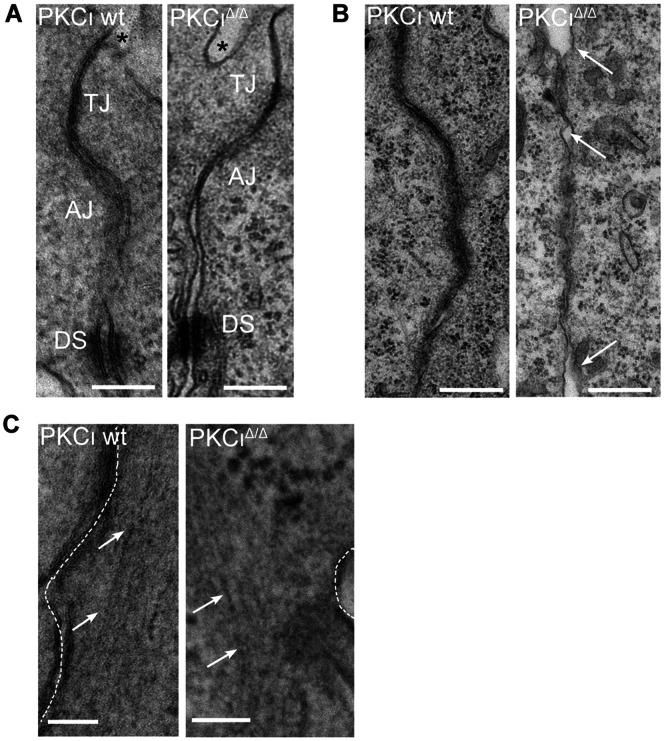
Electron microscopic analysis of PKCι deficient embryos. Embryos were isolated at E7.5. (A) Apical junctional complex (AJC) formation in wt and PKCι deficient (PKCι^Δ/Δ^) embryos was compared. Functional complexes are indicated: tight junction (TJ), adherens junctions (AJs) and desmosomes (DSs). The amniotic cavity is indicated by an asterix. (B) Arrows indicate intracellular space in the apicobasal membrane in PKCι wt and PKCι^Δ/Δ^ embryos. The orientation of the images is comparable to the images in A. (C) Arrows indicate the F-actin bundles parallel to the cell-membrane in wt and PKCι^Δ/Δ^ embryos. EM analysis was performed as described in Materials and methods. Representative micrographs are presented. Bar is equivalent to 200 micrometer (200 µm).

To further elucidate the structural organization of the cell-cell contacts, we analyzed wt and mutant embryonic tissue by using electron microscopy. Both genotypes showed a clear electron dense area at juxtaposed membranes located at the apical side defined as tight junctions (Figure 5A). AJs and desmosomes (DSs) were detected without identifying any difference among the genotypes (see Figure 5A). However, when focusing on the overall appearance, we observed a general tendency of mutants to possess a looser contact zone between individual cells. This was indicated by more open gaps within the extracellular space when compared with wt (exemplary shown in Figure 5B). At the same time we observed in sections of mutants that F-actin normally localized in tight bundles in proximity to the membrane appeared less tight organized. In addition, the distance between the bundles and the outer membrane seemed to be larger (Figure 5C). In summary the electron microscopic analysis provides further evidences for changes in the integrity of cell-cell contacts without PKCι.

### PKCζ is Able to Rescue the Early PKCι in vivo Function

PKCι and PKCζ are structurally highly related, suggesting shared *in vivo* functions and redundancy. We tested this assumption by establishing a PKCι^ζRes/+^ mouse line which expresses a chimeric PKCι/ζ protein. For this purpose we established a targeted (*knockin*) allele haboring an inserted PKCζ cDNA in the 2nd exon of the *Prkci* gene at codon position 29 gave rise to a fusion protein consisting of the first 28 amino acids of the PKCι protein followed by the remaining 564 amino acids of PKCζ which will be transcripted under PKCι conditions using its endogenous promotor. In addition we modified the 3′ UTR of the artificial transcript in such a way that the 3′ untranslated regulatory RNA sequences of the PKCι messenger RNA will be included thereby providing the same mRNA stability as the wt PKCι transcript (see Figure S1C and materials & methods).

**Figure 6:**
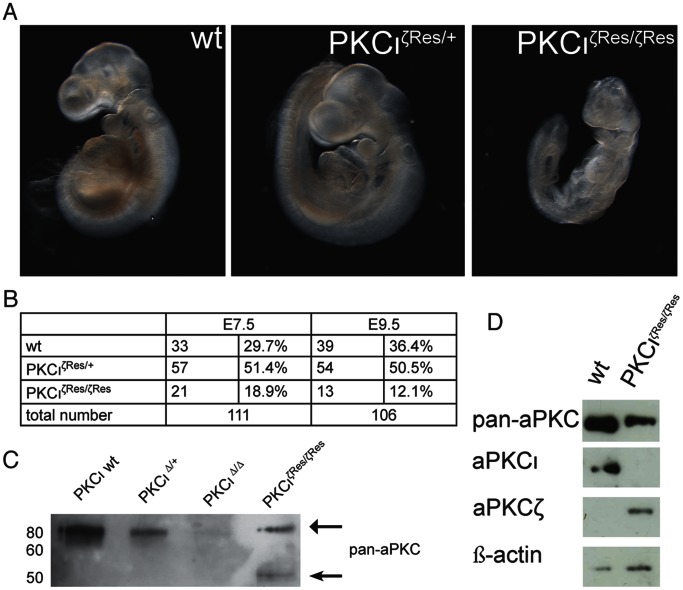
Overexpression of PKCζ rescues PKCι KO phenotype. (A) Comparison of the appearance of embryos, that are either wt, heterozygous for the rescue allele (PKCι^ζRes/+^) or homozygous for the rescue (PKCι^ζRes/ζRes^) allele embryos at E9.5. (B) Table containing the total numbers of analyzed embryos at day E7.5 and E9.5 and the corresponding percentage of the genotypes. (C) Western blot analysis of E7.5 embryos using an antibody against pan-aPKC. Each lane contains the total protein amount of a whole embryo. (D) Western blot analysis of day E9.5 embryos using indicated antibodies. Each lane contains adjusted (β-actin) protein amounts from single embryos.

To investigate whether homozygozity of the PKCι^ζRes^ allele is able to compensate for PKCι function during embryonic development, we first analyzed pups from matings of heterozygous carries for the PKCι^ζRes^ allele at E7.5. At that stage we did not detect any morphological changes within 111 embryos obtained from heterozygous intercrosses of this line. In addition, we were able to identify 21 homozygous embryos (18.9%) for the rescue allele (PKCι^ζRes/ζRes^) when we genotyped these embryos by PCR (Figure 6, table).

A Western Blot analysis of individual embryos revealed that wt and PKCι^Δ/+^ embryos showed an aPKC signal (using a pan aPKC antibody) whereas there was no detectable signal in the PKCι^Δ/Δ^. Interestingly in PKCι^ζRes/ζRes^ embryos an aPKC specific band was detectable including a 50 kDa fragment which is supposed to be a proteolytic product of PKCζ, proving the isoform specificity of the expressed protein (Figure 6C). We concluded that PKCζ expressed under endogenous PKCι condition is able to rescue the early PKCι phenotype. In contrast, we failed to identify any PKCι^ζRes/ζRes^ animals in 150 offspring derived from heterozygous crosses at postnatal day 21 indicating that PKCζ is not able to compensate for PKCι’s function during later stages of development (see table in Figure 6). To define the lethality of PKCι^ζRes/ζRes^ embryos, we screened several embryonic stages for morphologically divergent embryos. At E9.5, PKCι^ζRes/ζRes^ embryos were severely retarded in growth (Figure 6A). Western blot analysis revealed that PKCζ but not PKCι was expressed in the rescue embryos whereas PKCζ was not expressed in the wt (Figure 6D). Thus, PKCζ is able to compensate for PKCι *in vivo* function at E7.5 but not at later stages.

## Discussion

The aim of this study was to shed more light on early embryonic *in vivo* functions of PKCι in mouse. Earlier genetic studies in *C. elegans*, *Xenopus* and *Drosophila melanogaster* indicated a pivotal role of aPKC in the establishment and maintenance of polarity of oocytes and epithelial cells [48]–[51]. These findings were often related to the functional and physical association of aPKC to Par-3 and par6, which together form the polarity complex [31]. This ternary complex is conserved among species suggesting a related phenotype upon depletion of PKCι in mouse embryos. A report by Soloff et. al, 2004 described an embryonic lethal phenotype when the PKCι gene was knocked out by conventional gene targeting without a detailed developmental analysis. Both of our generated knock out mouse lines for PKCι showed an embryonic lethal phenotype with first morphological manifestations around E7.5. Mutant embryos appeared compressed and are characterized by a severely reduced amniotic cavity. Nevertheless a histological examination revealed still the existence of epithelial cells which was rather unexpected since all studies from other species identified alterations in epithelial cell layers. So far publications describing gene knockouts in mice of polarity related proteins do not exactly match phenotypically with the PKCι knockout. For example a conventional knock out of Cdc42, an established activator of aPKCs in the context of the Par-3/aPKC/Par6 complex, revealed a much earlier phenotype. Embryos deficient for Cdc42 showed first signs of alterations at E5.5 and were degraded at E6.5 [52]. Rac1, another small GTPase, which had been implicated many times as signal mediator of aPKC signaling also revealed an earlier phenotype in the knockout. In this case the main cause of lethality was linked to failures in the actin cytoskeletal reorganization combined with reduced motility and increased programmed cell death [53]. A complete knock out of Par-3 caused mid-gestational embryonic lethality (around E9.5) which was correlated to a defective cardiac development [54]. Unfortunately published data on a par6 knockout in mice are currently not available to our knowledge. One of the most similar knock outs published so far, when taking morphological criteria into account, is represented by the afadin knockout. Similar to the PKCι knockout morphological changes occurred at E7.5 and resembled what we saw in the PKCι deficiency (compressed embryo and small amniotic cavity) [55]. Nevertheless the described disorganized cell-cell junctions did not exactly match our observation. Interestingly afadin has been described as adapter protein connecting nectin to actin filaments [56] and to colocalize with Par-3/aPKC at AJs in the neuroepithelium of the developing mouse central nervous system [55]. Thus a similar function earlier during development is conceivable but further experiments are needed to underpin this hypothesis.

Our marker analysis revealed that PKCι-deficient embryos start to gastrulate and establish distinct anterior-posterior and dorso-ventral body axes at around E7.5–E7.75. However, axial elongation and mesoderm formation arrests around this stage and elaboration of all mesodermal tissues and organs fails. Absence of a functional cardiovascular system is the cause of the subsequent death. Given the fact that all embryonic tissue are affected our data suggest that PKCι is not regionally but cellularly required.

Applying the embryoid body formation assay we showed that mutant EBs failed to establish a single cavity instead they formed multiple small cavities. Nevertheless a proper formation of the basal lamina and the apical domain by various marker proteins could be detected (see Figure 3). Particular the correct localization of Par-3 and Mupp1 implied an established apical pole which to some content did not surprise since studies performed in *C. elegans* showed that loss of PKC-3 did not affect Par-3 localization either [56]. Interestingly EBs deficient for Cdc42 not only fail to form a single lumen cavity but also showed a complete loss of aPKC and Par-3 localization at the apical domain [57]. One reason for this difference might be that Cdc42 is not able to distinguish between the two aPKC isoforms and we showed that PKCζ is still present in PKCι deficient EBs. Thus we assume that depletion of Cdc42 resulted in a robust reduction of both aPKC activities causing a more severe phenotype. This finds support by the fact that overexpression of a dominant negative PKCζ version in wt EBs mimics the Cdc42 phenotype [57]. The formation of a single cavity has been described as a two-step process: first, multiple small lumen are formed in the periphery of the EB and within a second step smaller lumen are fused and remaining inner cells undergo apoptosis to clear the central lumen [42]. Our data suggest that the loss of PKCι did not interfere with the first step but disrupted the second. Whether PKCι acts on the fusion of the small cavities or the subsequently induced apoptosis or both is not solved yet but from our data it´s clear that PKCι deficiency caused a decrease of activated caspase-3 and less apoptotic cells in EBs.

An earlier study on the role of Par6 and aPKCs in Caco-2 cysts formation showed that down-regulation of either one of the proteins is causing multiple lumen formation as well. In this it was correlated to an impaired spindle orientation during morphogenesis [58]. These findings might represent an alternative explanation for the observed phenotype. However, we were not able to identify any indication for an impaired spindle formation, either in PKCι deficient embryos or in PKCι deficient EBs. Again, one likely reason for this difference might be that the knock-down of Par6 and both aPKCs is more dramatic than the loss of one single aPKC isoform. In this case the data would indicate compensatory functions in the context of spindle orientation in Caco-2 cysts.

The immunohistochemical analysis of E7.5 embryo sections revealed by the E-cadherin staining that the general polarity of the embryonic ectoderm is preserved in mutants. But it became apparent that the fine structure of the apical side is changed (indicated by its fuzzy appearance) when compared to the wt at higher magnification (Figure 4 C). As a possible explanation we found ZO-1, a tight junction protein, to be down-regulated and dislocated in epithelial cells deficient for PKCι. ZO-1 is believed to function as a junctional organizer by direct binding to tight junctional proteins (like occludin and claudins) and the actin cytoskeleton. Thus it could well be that the absence of ZO-1 in tight junction complexes could lead to a lack of proper connections of tight junctions to the actin cytoskeleton, causing a less organized actin belt at the apical side which leads to the initially observed fuzzy appearance. This hypothesis finds support by the EM analysis, which clearly indicate that F-actin filaments are less compact organized and the distance between the outer membrane and the F-actin filaments appears to be increased in mutants. Consequently we checked the ZO-1 localization in E7.5 rescue embryos. The ZO-1 protein appears to be localized at the apical site of the embryo comparable to what we see in the wt embryos (Figure S4). This result indicates that the low expression of PKCζ in the PKCι deficient background might be responsible for the loss of ZO-1 localization. Nevertheless a conventional ZO-1 knockout displayed a later phenotype mainly associated with defect in angiogenesis and increased apoptosis [59]. One plausible explanation for this might be the existence of compensatory mechanisms in the ZO-1 knock out, which do not exist in the PKCι deficiency. As for example the remaining ZO-2/3 proteins might function redundant when ZO-1 was deleted whereas a knockout of a crucial downstream kinase cannot be compensated for in this system.

When analyzing aPKCs cellular localization (Figure 4B) we mainly detected aPKC at the apical side, representing the expected pattern for aPKC in epithelial cell layer[60]–[62]. To our surprise a nearly unchanged pattern was detected in mutants as well, indicating that either aPKCs share overlapping expression domains or PKCζ becomes up-regulated in mutants. We favor the first since neither a semi-quantitative PCR nor a Western Blot analysis of E7.5 PKCι^Δ**/+**^ and E8.5 PKCι^Δ/Δ^ was able to identify an up-regulation of PKCζ (Figure S3). Thus, we assume that PKCζ can potentially take part at the predicted *in vivo* function of aPKCs with regard on the establishment and maintenance of polarity due to its cellular localization, thereby might compensate for PKCι deficiency. In addition the increased cellular localization pattern of PKCι, identified by a specific antibody, implied that PKCι might fulfill other *in vivo* functions than the ones predicted by its association to the Par-3 and par6 proteins.

When PKCζ was expressed under the same spatial and temporal conditions like PKCι (PKCι^ζRes/ζRes^ allele) we were not able to detect embryos at E7.5 displaying the morphological changes described earlier. We concluded that PKCζ is able to compensate for PKCι *in vivo* function at E7.5 when expressed at higher levels than in the wt. Thus the originally defined PKCι specific *in vivo* function at E7.5 represents an aPKC signaling pathway which became PKCι specific through a very tight transcriptional control. However, besides no visible difference between wt and rescue embryos at E7.5 and E8.5 the PKCι^ζRes^ allele is not able to compensate for PKCι functions at E9.5 since embryos homozygous for this allele showed a clear growth retardation resulting in lethality one to two days later. This points to the fact that later during development PKCι *in vivo* function exists which are not redundant among the aPKC subfamily and could therefore be described as isoform-specific. Nevertheless at this point we are not able to exclude that an artificial overexpression of PKCζ has toxic effects for the embryo or that the observe proteolytic 50 kDa fragment (Figure 6D) which had been linked to apoptotic process [63] causes the embryonic lethality. Worth mentioning at this point is the fact that within an earlier publication we have reported a week but detectable PKCζ expression by western blotting in wt E9.5 embryos [15] whereas no PKCζ protein was detected in this study. One possible explanation for this discrepancy might be that different amounts of protein were loaded since in this study we used extracts from single embryos whereas in the earlier publication pools of embryos served as starting material. Other reasons might be a different batch of the antibody as well as variations in the exposure time. In any case expression levels of PKCζ could be stated as lower when compared to PKCι at the same embryonic stage.

In summary we showed that the depletion of PKCι caused embryonic lethality with the earliest time-point of visible morphological changes at E7.5. To our surprise we still detected a strong staining for aPKC at the apical membrane in PKCι deficient embryos indicating that a reasonable amount of PKCζ still localize to this area. This per se might be a reason why the basal to apical cell architecture in PKCι deficient embryos was still conserved. In addition it also identified a possible PKCζ function in this context by its very precise localization which by far was not expected based on expression data earlier published. As a result of PKCι deficiency we identified a severe down-regulation of ZO-1 which could explain the subtle alteration in the cellular structure. Nevertheless the cellular mechanism of PKCι *in vivo* function at E7.5 remained unsolved. However, when we expressed PKCζ in PKCι deficient embryos the initial lethal phenotype at E7.5 could be rescued. This identifies the aPKCs as a very redundant kinase subfamily in which transcriptional as well as spatial cues regulate its isoform specificity.

## Materials and Methods

### Generation of Mutant Prkci Alleles

To clone the mouse Prkci locus a 129/Ola genomic cosmid library (obtained from the Resourcenzentrum, Berlin, Germany) was screened using a full length mouse cDNA as a probe. Several cosmid clones were identified and further purified. One of those containing the genomic 5′prime part of the gene was selected for further cloning. All further cloning strategies followed standard procedures described in [64] and [65]. To generate the following targeting constructs for the PKCι gene a 10.9 kb genomic EcoRI fragment, including the 2nd exon (corresponding to nucleotides 110–233 of the published murine PKCι cDNA), was subcloned into a bluescript backbone. Using this genomic DNA fragment the conventional targeting vector was generated by inserting an independent neo-cassette (derived from pMC1neoPolyA from Stratagene) into a Sal I restriction site, which was introduced into the 2nd exon by site directed mutagenesis. As a consequence of this insertion the transcription of the PKCι gene is supposed to be abrogated. Based on the same genomic DNA backbone the conditional targeting vector was generated by introducing a single loxP site into a unique EcoRV restriction site 3.3 kb located upstream of the 2nd exon subsequently followed by an insertion of a loxP-flanked neo-cassette (derived from pL2-neo8, kindly provided originally by Klaus Rajewskýs laboratory, Cologne, Germany) into a BglII restriction site 1.5 kb downstream of the 2nd exon. Upon incubation with the Cre recombinase this will lead to a deletion of a ∼ 6.0 kb genomic DNA fragment including the 2nd exon, thereby causing a frame shift within the transcript, leading to a nonsense mRNA. The functionality of this allele has been proven by a sufficient number of tissue specific Cre mediated deletions in mice (see for example [25]).

In order to subclone the PKCι rescue targeting vector again the 10.9 kb genomic EcoRI fragment served as the starting material, including the introduced SalI restriction site within the 2nd exon. A mouse PKCζ cDNA fragment (consisting of the cDNA sequence for codon 29 till the STOP codon followed by the complete 3′ UTR sequence of the PKCι transcript) was inserted in frame into the SalI site which corresponds to codon 29 of the PKCι gene. In addition a loxP-flanked neo-cassette for later selection purposes was subcloned right after the PKCι 3′ UTR. The resulting PKCι rescue allele expresses a fusion protein consisting of the first 28 amino acids of PKCι and the remaining 564 amino acids of PKCζ under the control of the endogenous PKCι promotor and the PKCι 5′ and 3′UTRs.

All three targeting vectors were linearized before electroporation. Applied ES cells were from the substrain E14.1 background (129/Ola), kindly provided by Ralf Kühn, Institut for Genetics, Cologne, Germany. For each electroporation 2×96 G418-resistant ES cell clones were screened for a homologous recombination event of the targeting vector by Southern blot analysis [66]. The observed targeting frequency was about 30–40%, respectively. For each targeting event at least 2 ES cell clones were further characterized by Southern Blotting using indicated 5′and 3′probes and the internal neo cassette (see Figure S1) to check for a correct integration of the corresponding targeting vector into the PKCι gene locus. Verified ES cell clones were further used for injections into NMRI blastocysts. Chimeric males were obtained for each construct and subsequently mated to NMRI females to test for germ-line transmission. Each targeted mutation gave rise to F1 heterozygous males which were then further used for establishing the corresponding mouse lines for phenotypic analysis.

### Mice and Genotyping

Mice carrying a null allele of *Prkci* and the PKCι^ζRes^ allele were maintained on an outbred (NMRI) background. For timed pregnancies, vaginal plugs were checked in the morning after mating; noon was taken as embryonic day (E) 0.5. Pregnant females were sacrificed by cervical dislocation; embryos were harvested in phosphate-buffered saline, decapitated, fixed in 4% paraformaldehyde overnight, and stored in 100% methanol at −20°C before further use. Genomic DNA prepared from yolk sacs or tail biopsies was used for genotyping by polymerase chain reaction (PCR). Primer used: Iota_del_forward: ACT AAG CAT TGC CTG GCA TC; Iota_del_reverse: AAT TGT TCA TGT TCA ACA CTG CT; Iota_Ex2_forward: TGG AAG GAA AGG AA G TGT GC; Iota_Ex2_reverse: GGT GAA CGG CTG CTC ATT; Iota_Fus_forward: AG C CCC AGA TCA CAG ATG AC; Iota_Fus_reverse: CTC GAA TCC TGC CTC CTG A AG.

### Ethics Statement

All animal work conducted for this study was approved by The Norwegian Institute of public health and performed according to Norwegian legislation.

### Hematoxylin and Eosin Staining of Embryonic Sections

For histological analysis, wt or aPKC mutant embryos were fixed in 4% paraformaldehyde and embedded in paraffin wax. Sections of 4 µm were processed for cytohistochemistry after de-paraffinization. After rehydration the section were incubated in 1× Gill’s Hematoxylin (haematoxylin.

6.0 g, aluminum sulphate 42.0 g, ethylene glycol 269 ml, citric acid 1.4 g, sodium iodate 0.6 g, distilled water 680 ml) for 5 minutes. After washing the samples were incubated in acid alcohol (1% HCl in 70% EtOH) and washed again. After incubation in ammonia water the samples were rinsed and incubated in eosin Y (Sigma-Aldrich, HT110132-1L) for 1 minute. Before mounting with a xylene-based mounting medium cells were dehydrated. Embryos were documented using Zeiss Lumar V.12 with AxioCam HRc camera.

### Whole Mount RNA in situ Analysis

Whole-mount *in situ* hybridization on E8.5 embryos was performed following a standard procedure with digoxigenin-labeled antisense riboprobes [67].

### Embryoid Body Culture and Processing

The ES cell line used for this study were wt E14 and PKCι^Δ/Δ^ ES cells [43]. Wt and PKCι^Δ/Δ^ ES cells were cultured on mitomycin C- treated STO cells. EB formation was initiated from ES cell aggregates in suspension culture as described previously [68]. For immunofluorescence, EBs were collected into 15-ml conical tubes, allowed to sediment by gravity, washed once in phosphate-buffered saline (PBS), and fixed with 3% paraformaldehyde. EBs were then embedded in OCT compound, sectioned on a Leica cryostat and immunostained for antigens of interest [68]. F-actin was stained with rhodamine-phalloidin (Molecular Probes). Nuclei were counterstained with DAPI. Slides were examined with a Nikon inverted fluorescence microscope (Eclipse TE 2000) and digital images were acquired with a cooled CCD camera (Hamamatsu) controlled by IP Lab 4.0 software (Scanalytics). For immunoblotting, EBs were washed 3 times in PBS and lysed in SDS lysis buffer (50 mM Tris, pH 7.4, 150 mM NaCl, 1% SDS) containing protease and phosphatase inhibitor cocktails. Protein concentrations were determined using BCA reagents (Pierce). Proteins of equal loads were resolved by SDS-PAGE and then transferred onto PVDF membranes, which were blocked with 5% nonfat dry milk. After incubation with primary antibodies, specific signals were detected with HRP-conjugated secondary antibodies and ECL reagents. Antibodies used: PKCζ rabbit polyclonal antibody (Cat# 07-264) and GAPDH mouse monoclonal antibody were purchased from Millipore. Perlecan rat monoclonal antibody was purchased from Santa Cruz Biotechnology, Inc. Cleaved caspase-3 rabbit polyclonal antibody was bought from Cell Signaling Technology. Actin polyclonal antibody was bought from Sigma.

### Immunohistochemistry and Imaging Analysis

For Immunohistological analysis, wt and aPKC mutant embryos were fixed in 4% paraformaldehyde and embedded in paraffin wax following standard protocols. Sections of 4 µm were processed for immunohistochemistry. Antigen retrieval was performed after de-waxing to enhance staining. Sections were then incubated with 5% fetal calf serum for 1 hour, and then washed three times with sterile PBS (pH 7.5) prior to overnight incubation with the appropriate primary antibodies at optimal dilutions (PKCζ, SantaCruz, 1∶200; PKCι, 1∶5000; PKCζ, 1∶7500 [47]; claudin, Zymed, 1∶500; occludin, Zymed, 1∶500; ZO-1, Zymed, 1∶100; cleaved caspase-3, Cell Signaling, 1∶500, phistone 3, Cell Signaling, 1∶500, E-cadherin, Santa Cruz, 1∶500). The secondary antibody, AlexaFluor 647 conjugated anti-rabbit antibody was bought from Invitrogen®, was used in a 1∶1000 dilution and overnight incubation. The section were then mounted in Prolong Gold with DAPI reagent from Invitrogen® (#P36930). Images were taken with the Zeiss LSM 510 Meta invert microscope equipped with a Zeiss LSM laser module and a AxioCam HMR digital camera.

### Electron Microscopy

The embryos were fixed in 2% glutaraldehyde in 0.1 M sodium cacodylate buffer. After washing, specimens were rinsed twice for 10 min in 0.1 M sodium cacodylate buffer and postfixed with 2% osmiumtetroxide (OsO4) containing 1.5% potassium ferric cyanide ((K3Fe(CN)6)) for 1 h. Following extensive rinsing with water, dehydration with an alcohol series was performed. Specimens were placed in 100% alcohol and Epon 1∶1 overnight and then embedded in pure Epon. Ultrathin sections were cut with a Leica microtome for TEM microscopy and placed on carbon coated copper grids. Images were taken with a Philips CM 100 transmission electron microscope (TEM) at various magnifications.

### Western Blot Analysis of Embryos

Embryos were collected isolated at either E7.5 or E9.5 from the mother animal. All extraembryonic tissue was removed before lysing the whole embryo in protein extraction buffer for 20 min. on ice (50 mM Tris/HCl, pH 7.5–8.0; 2 mM EDTA, pH7.0; 10 mM EGTA, pH 7.0; 0.1% TritonX-100, 3% β-mercaptoethanol) supplemented with Proteinase Inhibitor Cocktail (Sigma, #P-2714). A 31 gauge syringe was used to disrupt the tissue. The samples were centrifuged at 8.000 rpm for 8 min and the supernatant was transferred into a new tube before supplementing protein sample loading buffer and boiling for 5 min. The samples analyzed on a 10% SDS- polyacrylamide gel and blotted onto BioRad nitrocellulose membranes. Unless stated otherwise the protein amounts were adjusted based on the β-actin levels. The membranes were blocked in 5% nonfat dry milk in TBST for 1 h at room temperature. Incubation with the primary antibody overnight in TBST at 4°C. The following antibodies were used: anti-PKCζ (Santa Cruz, C-20), anti-PKCι 1∶10.000 [47], anti-PKCζ 1∶5000 [47] and anti-β-actin (Santa Cruz, sc-47778 HRP).

### Semi-quantitative RT-PCR Analysis

Embryos were isolated at appropriate stage and directly lysed in TRIZOL reagent from Invitrogen®. The RNA isolation was performed according to the TRIZOL manual. The reverse transcription to obtain cDNA was performed using the iSCRIPT- cDNA synthesis kit from Bio-RAD. For the expression analysis the HotStar Taq PLus DNA Polymerase from Qiagen in combination with following primers was used: ZO-1 (forward-primer AGC TGT TTC CTC CAT TGC TG, reverse-primer: GAG ATG TTT ATG CGG ACG GT), PKC ζ (forward-primer: GCC TCC CTT CCA GCC CCA GA, reverse-primer: CAC GGA CT C CT C AGC AGA CAG CA) and PKCι (forward-primer: AGG AAC GAT TGG GTT GTC AC, reverse-primer: GGC AAG CAG AAT CAG ACA CA). All primers were designed with an optimal annealing temperature at 60°C and a amplicon size of around 300 bp.

## Supporting Information

Figure S1 Targeting of the PKCι locus in mice. Schematic representation of the chosen targeting strategies is shown. Restriction maps indicating for each targeting approach the relevant restriction sites of the wild type and mutant locus are shown (B, BamHI; Bg, BglII; E, EcoRI; EV, EcoRV; H, HindIII; K, KpnI). The black box represents the second exon of the PKCι gene which was used for all targeted modifications. The white boxes below the mutant alleles indicate the 5′*HindIII*-*EcoRI* RFLP probe (5′probe) and the 3′*KpnI*-*EcoRI* RFLP probe (3′probe) used for later southern blot analysis. Generated targeted alleles are listed as following: A) *conventional knockout* - *PKCι* WT locus, the targeting construct (harboring an inserted neo cassette into the 2nd exon) and the mutant *PKCι* locus (*PKCι Neo allele)* after homologous recombination are shown. B) *conditional knockout* - *PKCι* WT locus, the targeting construct (containing a single LoxP site 5′ and a floxed neo cassette 3′ of the 2nd exon) and the mutant *PKCι* locus (*PKCι floxed allele)* after homologous recombination and subsequent deletion via Cre recombinase are shown. C) *rescue knockin* (*PKCι ζRes allele*) - *PKCι* WT locus, the targeting construct (containing an inserted PKCζ cDNA into the coding sequence of the 2nd exon followed by a floxed neo cassette) and the mutant *PKCι* locus (*PKCι ζRes allele)* after homologous recombination and subsequent deletion of the neo cassette via Cre recombinase are shown. D) Southern blot analysis of embryonic stem cell clones after G418 selection (*PKCι Neo allele; PKCι floxed allele)* and heterozygous offspring from intercrosses of the *PKCι Δ allele* and *PKCι ζRes allele.* Top left; BamHI RFLP using the 5′probe identifies 2 clones of the conventional targeting by a 6.5 kb band whereas the 8.0 kb band represents the wt band, top right: HindIII RFLP using the 5 ´probe identifies one targeted ES cell clone for the floxed allele (3.0 kb band repesents the wt whereas the 2.0 kb band indicates a mutant), bottom left; the *PKCι Δ allele* was characterized by EcoRI using the 3′probe resulting in an 11.0 kb wt and a 3.2 kb mutant.
(EPS)

Figure S2 Proliferation and apoptosis in PKCι mutant embryos. E7.5 embryos were embedded in paraffin and then sectioned into a 5 µm thick slices. Immunofluorescence analysis were performed using the indicated antibodies. Markers for either apoptosis (cleaved caspase-3, 1∶500) or proliferation (phospho-histone-3, 1∶500) were used for immunofluorescence analysis of PKCι^Δ/Δ^ embryos or wt. All analysis were done using the Zeiss LSM 510 confocal microscope. Scale bar: 100 µm
(TIFF)

Figure S3 Protein expression and protein levels analysis in wt and PKCι deficient embryos. (A) Expression analysis of indicated proteins in wt and PKCι^Δ/Δ^ embryos at stage E7.5. (B) WB analysis of WT and PKCι^Δ/Δ^ embryos at stage E7.5 and E8.0 using antibodies as indicated. Results of the aPKC blot are shown with different exposure times: 2 min (lower lane) and 5 min (upper lane).
(TIFF)

Figure S4 Localization of ZO-1 at the apical domain is re-established in PKCι^ζRes/ζRes^ embryos. Analysis were performed using a specific ZO-1 antibody. Paraffin sections showing the localization of the Z0-1 in the wt and PKCtSRes/i;Res embryo. Scale bars: l00 µm.
(TIFF)
