# Supplementary figures and images for: Phenotypical Analysis of Atypical PKCs In Vivo Function Display a Compensatory System at Mouse Embryonic Day 7.5
Source: PLoS One. 2013 May 14;8(5):e62756. doi: 10.1371/journal.pone.0062756 (PMC3653893; doi:10.1371/journal.pone.0062756)

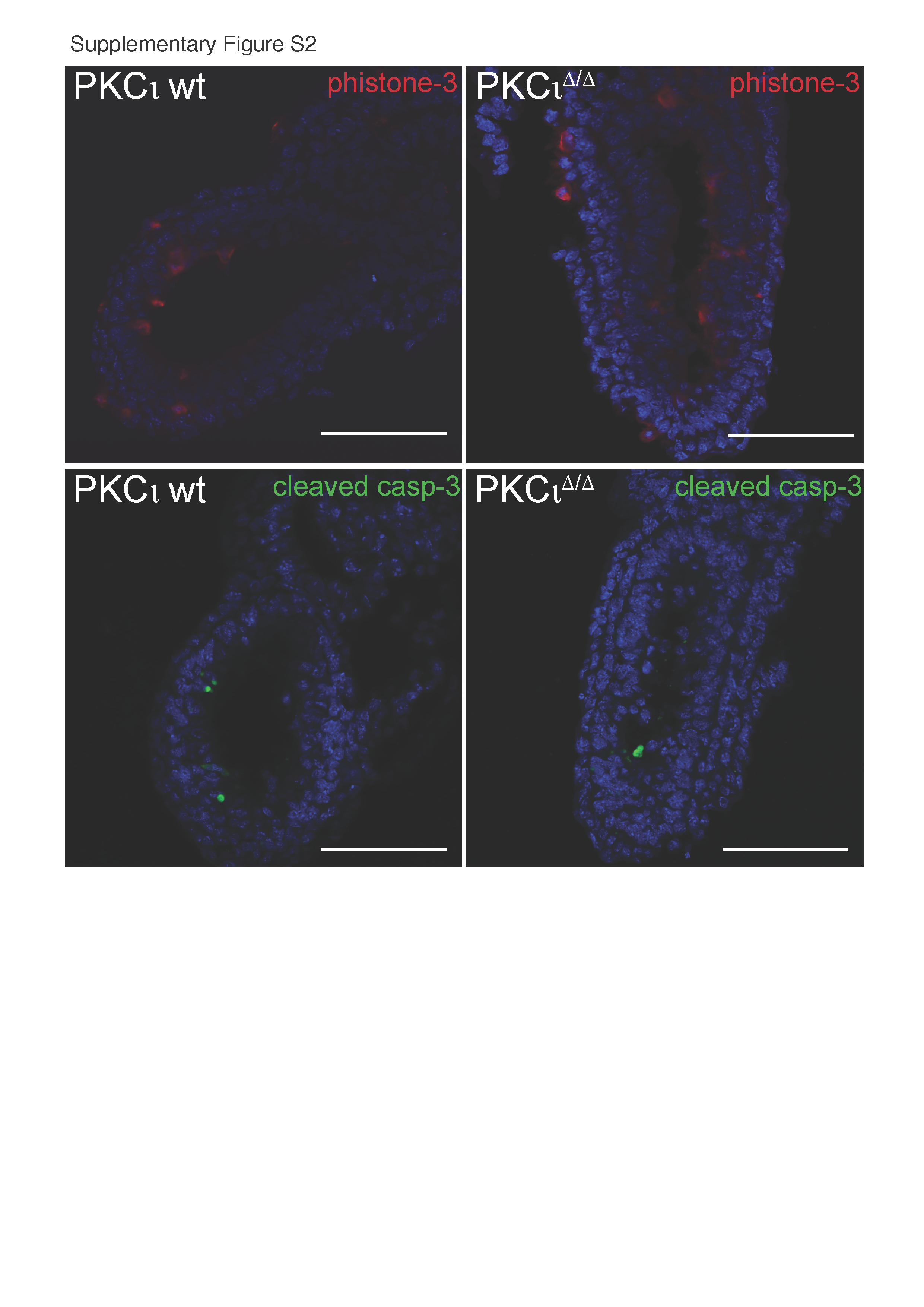

Supplement: Figure S2 — Proliferation and apoptosis in PKCι mutant embryos. E7.5 embryos were embedded in paraffin and then sectioned into a 5 µm thick slices. Immunofluorescence analysis were performed using the indicated antibodies. Markers for either apoptosis (cleaved caspase-3, 1∶500) or proliferation (phospho-histone-3, 1∶500) were used for immunofluorescence analysis of PKCιΔ/Δ embryos or wt. All analysis were done using the Zeiss LSM 510 confocal microscope. Scale bar: 100 µm (TIFF) [file pone.0062756.s002.tiff]

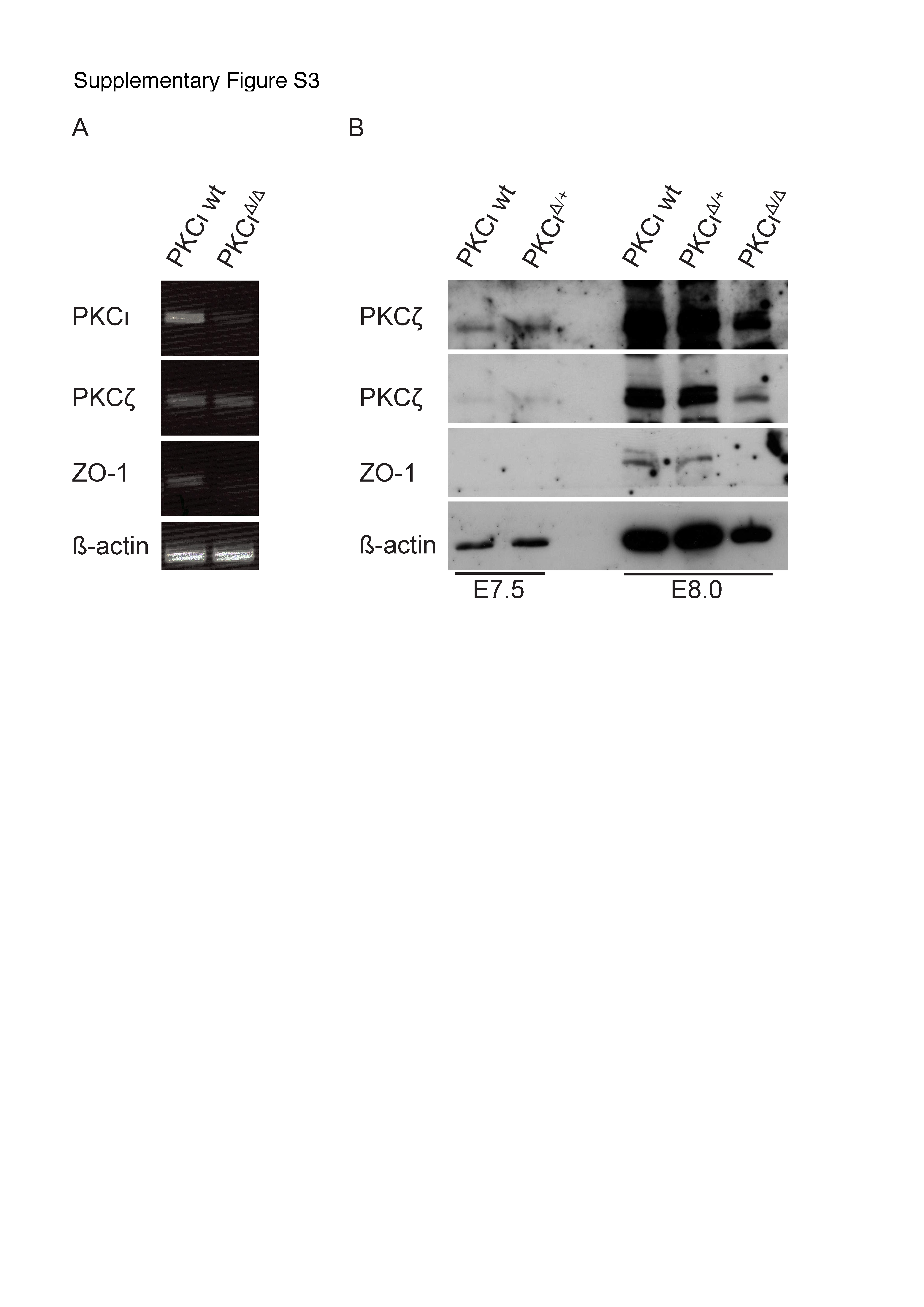

Supplement: Figure S3 — Protein expression and protein levels analysis in wt and PKCι deficient embryos. (A) Expression analysis of indicated proteins in wt and PKCιΔ/Δ embryos at stage E7.5. (B) WB analysis of WT and PKCιΔ/Δ embryos at stage E7.5 and E8.0 using antibodies as indicated. Results of the aPKC blot are shown with different exposure times: 2 min (lower lane) and 5 min (upper lane). (TIFF) [file pone.0062756.s003.tiff]

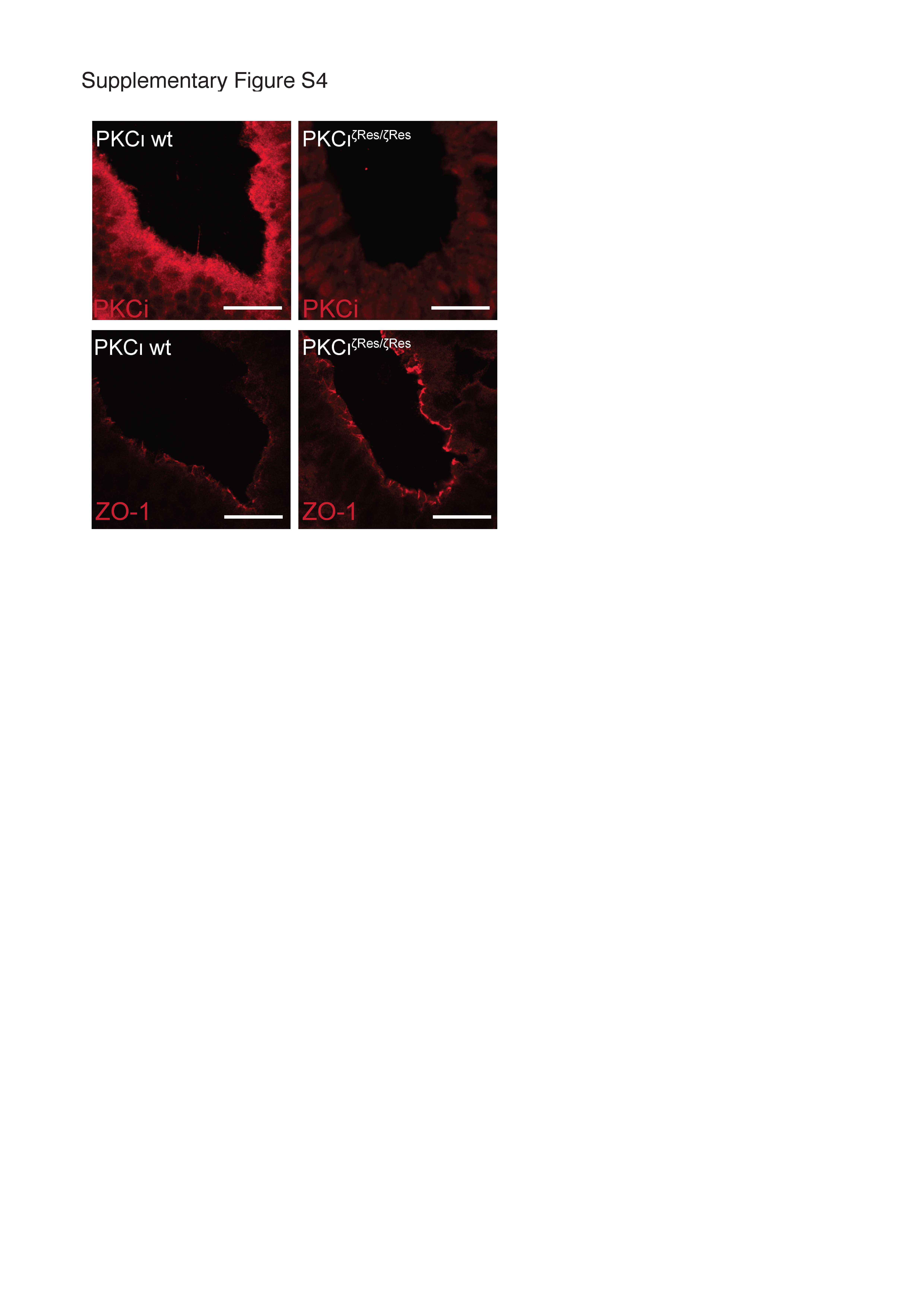

Supplement: Figure S4 — Localization of ZO-1 at the apical domain is re-established in PKCιζRes/ζRes embryos. Analysis were performed using a specific ZO-1 antibody. Paraffin sections showing the localization of the Z0-1 in the wt and PKCtSRes/i;Res embryo. Scale bars: l00 µm. (TIFF) [file pone.0062756.s004.tiff]
